# *Sarandibrinus*, a new genus of Saprininae subfamily from Madagascar (Coleoptera, Histeridae) (Second contribution to the knowledge of the Histeridae of Madagascar)

**DOI:** 10.3897/zookeys.427.4799

**Published:** 2014-07-22

**Authors:** Tomáš Lackner, Yves Gomy

**Affiliations:** 1 Czech University of Life Sciences, Faculty of Forestry and Wood Sciences, Department of Forest Protection and Entomology, Kamýcká 1176, CZ-165 21 Praha 6 – Suchdol,Czech Republic; 2 2 Boulevard Victor Hugo, 58000 Nevers, France

**Keywords:** *Sarandibrinus*, Coleoptera, Histeridae, Saprininae, taxonomy, Madagascar

## Abstract

*Sarandibrinus araceliae*, a new genus and species of the Saprininae subfamily is described from southern Madagascar. The new taxon exhibits autapomorphic characters for the Saprininae subfamily and is unusual especially for its large and deep prosternal foveae and the shape of spiculum gastrale. The description is accompanied by color habitus images, SEM micrographs, mouthparts and antenna line drawings and drawings of the male genitalia. Key to the genera of the Saprininae of Madagascar and the adjacent archipelagos is given. *Hypocaccus* (*Baeckmanniolus*) *rubiciliae* (Lewis, 1899) is newly reported from Madagascar and *Hypocaccus* (*Nessus*) *perparvulus* (Desbordes, 1916) is new to Mauritius.

## Abstract (fr)

*Sarandibrinus araceliae*, nouvelle espèce d'un genre nouveau de la sous-famille des Saprininae, est décrite du sud de Madagascar. Le nouveau taxon présente des caractères autapomorphiques pour les Saprininae spécialement par la présence de grandes fovéoles prosternales et par la forme de son spiculum gastrale. La description est accompagnée de photographies d'habitus en couleurs, de micro-photographies au microscope à balayage (SEM) et de dessins des antennes, des pièces buccales et des genitalia. Une clé des genres de Saprininae de Madagascar et des archipels voisins est donnée. *Hypocaccus* (*Baeckmanniolus*) *rubiciliae* (Lewis, 1899) est signalé comme nouveau pour Madagascar et *Hypocaccus* (*Nessus*) *perparvulus* (Desbordes, 1916), comme nouveau pour l'île Maurice.

## Introduction

With only 7 genera and 22 species (see Table 1) of the Saprininae subfamily reported from the fourth largest island of the Earth, Madagascar, including the adjacent archipelagos of Comoros, Mascarene Islands and Seychelles (Mazur 2011, Lackner and Gomy 2013 and present paper), this family is unquestionably poorly represented in the area in question. This statement can be put into perspective with the nearby-situated continent of Africa, which houses 26 genera with 100+ species of the Saprininae (Mazur 2011 and unpublished data). Among the Malagasy taxa – some of which are currently still being studied – only 6 species and one genus currently known are endemic to Madagascar (Mazur 2011; Lackner and Gomy 2013; present contribution). The other taxa occurring on the archipelagos or on Madagascar show either traits of regional distribution (western Indian Ocean), or are of different biogeographic origins (Afrotropical, Mediterranean, Oriental and even Neotropical). Some of these species have probably been introduced to Madagascar and the nearby Mascarene Islands, Comoros or Seychelles by human activity during recent centuries (Gomy 1983). The recent description of the first endemic genus *Malagasyprinus* (Lackner and Gomy 2013) containing three endemic and allopatric species emphasized the uniqueness of Malagasy Saprininae. With the description of another autapomorphic genus below, we further document the distinctness of the Malagasy Saprininae, which has been largely unrecognized hitherto.

**Table 1.** Distribution of the members of the Saprininae subfamily on Madagascar (Mad), Seychelles (Sey), Comoros (Com), and Mascarene (Masc) archipelagos. Taxa marked with * are endemic for the area in question and taxa marked with † are new for the islands/archipelagos in question.

		Mad	Sey	Com	Masc
	HISTERIDAE Gyllenhal, 1808				
	Saprininae Blanchard, 1845				
1	*Euspilotus (Hesperosaprinus) modestus* (Erichson, 1834)	-	-	-	+
2	*Euspilotus (Neosaprinus) rubriculus* (Marseul, 1855)	-	-	-	+
3	*Gnathoncus* sp.	+	-	-	-
4	*Hypocacculus* (s. str.) *metallescens* (Erichson, 1834)	-	-	-	+
5	Hypocaccus (Nessus) perparvulus*† (Desbordes, 1916)	+	-	-	+
6	*Hypocaccus (Nessus) vulturnus* (Reichardt, 1932)	-	-	-	+
7	*Hypocaccus (Nessus)* sp.	+	-	-	-
8	*Hypocaccus* (s. str.) *brasiliensis* (Paykull, 1811)	+	+	-	+
9	*Hypocaccus (Baeckmanniolus) disjunctus* (Marseul, 1855)*	+	+	+	-
10	*Hypocaccus* (s. str.) sp.	+	-	-	-
11	*Hypocaccus (Baeckmanniolus) rubiciliae* (Lewis, 1899)†	+	-	-	-
12	Malagasyprinus caeruleatus* (Lewis, 1905)	+	-	-	-
13	Malagasyprinus diana* (Lackner & Gomy, 2013)	+	-	-	-
14	Malagasyprinus perrieri* (Lackner & Gomy, 2013)	+	-	-	-
15	*Saprinus* (s. str.) *basalis* Fairmaire, 1898	+	-	-	-
16	*Saprinus* (s. str.) *chalcites* (Illiger, 1807)	-	-	-	+
17	*Saprinus* (s. str.) *cupreus* Erichson, 1834	+	-	-	-
18	*Saprinus (Phaonius) erichsonii* (Marseul, 1855)	+	+	-	+
19	*Saprinus* (s. str.) fulgidicollis* Marseul, 1855	+	-	-	-
20	*Saprinus* (s. str.) sp.	+	-	-	-
21	*Saprinus* (s. str.) *spendens* (Paykull, 1811)	+	+	-	-
22	Sarandibrinus araceliae* sp. n.	+	-	-	-
	Total	17	4	1	8

## Material and methods

All dry-mounted specimens were relaxed in warm water for several hours or overnight, depending on the body size. After removal from original cards, the beetles were side-mounted on triangular points and observed under a Nikon 102 stereoscopic microscope with diffused light. Some structures were studied using methods described by Ôhara (1994): the mouthparts, antenna and male genitalia were macerated in a hot 10% KOH solution for about 15 minutes, cleared in 80% alcohol, macerated in lactic acid with fuchsine, incubated at 60 °C for two hours, and subsequently transferred into a mixture of glacial acetic acid 1 part and methyl salicylate 1 part heated at 60 °C for 15 minutes and cleared in xylene. Specimens were then observed in α-terpineol in a small glass dish. Digital photographs of the male terminalia and antenna were taken by a Nikon 4500 Coolpix camera and edited in Adobe Photoshop CS5. Based on the photographs or direct observations, the mouthpars, genitalia and antennal structures were drawn using a light-box Hakuba klv-7000. SEM micrographs were taken at the Laboratory of the Electron Microscopy at the Faculty of Biology, Charles University, Prague, Czech Republic. Habitus photographs were made by F. Slamka (Bratislava, Slovakia). All available specimens were measured with an ocular micrometer. Beetle terminology follows that of Ôhara (1994) and Lackner (2010). Separate lines of the same label are marked by slash (/). The following acronyms of museums and private collections are used throughout the text:

CAS California Academy of Sciences, San Francisco, USA (D. Kavanaugh);

CYG Yves Gomy collection, Nevers, France;

TLAN Tomáš Lackner collection, temporarily housed at NCB Naturalis, Leiden, Netherlands.

**Abbreviations used in measurements:**

PEL Length between anterior angles of pronotum and apices of elytra;

APW Width between anterior angles of pronotum;

PPW Width between posterior angles of pronotum;

EL Length of elytron along sutural line;

EW Maximal width between outer margins of elytra.

## Taxonomy

### Sarandibrinus gen. n.

Taxon classificationAnimaliaColeopteraHisteridae

http://zoobank.org/DFCA5BA0-849E-47D6-8BF4-0C676960CA3A

#### Type species.

*Sarandibrinus araceliae* sp. n.

#### Diagnosis.

Small, castaneous, shining Saprininae; basal half of elytra glabrous, apical half covered with rugulose-lacunose punctation. Head comparatively large, frontal and supra-orbital striae absent, sensory structures of antennal club in form a single ball-shaped vesicle situated on internal distal side of the antenna under the apical slit-like orifice, pronotum wholly punctate, pronotal hypomeron ciliate; prosternal foveae large, round and deep. Meso- and metaventrite as well as first visible abdominal ventrite wholly punctate; spiculum gastrale only slightly constricted laterally instead of possessing a clear ‘head’ and ‘stem’ (sensu Caterino and Tishechkin 2013).

#### Differential diagnosis.

By the absent frontal and supra-orbital striae this taxon can be confused with two other sympatric genera: *Gnathoncus* Jacquelin du Val, 1858 (differing from it by the cuticular colour as well as presence of prosternal foveae) or *Euspilotus* Lewis, 1907, differing from it likewise by the metallic dorsum, ciliate pronotal epipleuron (both species of *Euspilotus* known from the region have glabrous pronotal hypomeron) or coarsely punctate frontal disk, with punctures forming elongate rugae (both *Euspilotus* species known from the region have their frontal disks covered with scattered punctures, never forming elongate rugae. The best differentiating character between the three taxa is probably the number of vesicles inside the antennal club: *Gnathoncus* possesses five, *Euspilotus* two or three, respectively, while *Sarandibrinus* has only one vesicle. The single vesicle character is present also in the sympatric genera *Hypocaccus*, *Hypocacculus* and *Malagasyprinus*, but the vesicle is pear-shaped vs. ball shaped in *Sarandibrinus*. *Hypocaccus*, *Hypocacculus* and *Malagasyprinus* furthermore, possess frontal and supraorbital striae, while *Sarandibrinus* lacks both. By the large oblique and deep prosternal foveae this taxon can be confused with the recently described genus *Malagasyprinus* Lackner & Gomy, 2013, differing from it by the cuticular colour (castaneous vs. dark blue/green) as well as absence of frontal and supra-orbital striae (*Malagasyprinus* possesses supra-orbital striae and its frontal stria is widely interrupted and prolonged onto clypeus). See also Key to the genera of the Saprininae from Madagascar (below).

#### Biology.

The series of the new taxon was collected by sifting the litter in spiny forest (thicket) as well as by flight intercept (or yellow pan, Malaise) traps in desert scrub forest.

#### Distribution.

Madagascar, Toliara Province (Fig. 22).

#### Etymology.

The name of this new genus has been formed using the word “Sarandib” – one of the ancient names given by Arabs to the far-flung island that later became Madagascar and “*rinus*” – the two final syllabus of the name “*Saprinus*” to demonstrate its position as belonging to the Saprininae subfamily.

### Sarandibrinus araceliae sp. n.

Taxon classificationAnimaliaColeopteraHisteridae

http://zoobank.org/D5441788-EE72-4883-80BD-C9BB89099526

**Figures 1–2.**
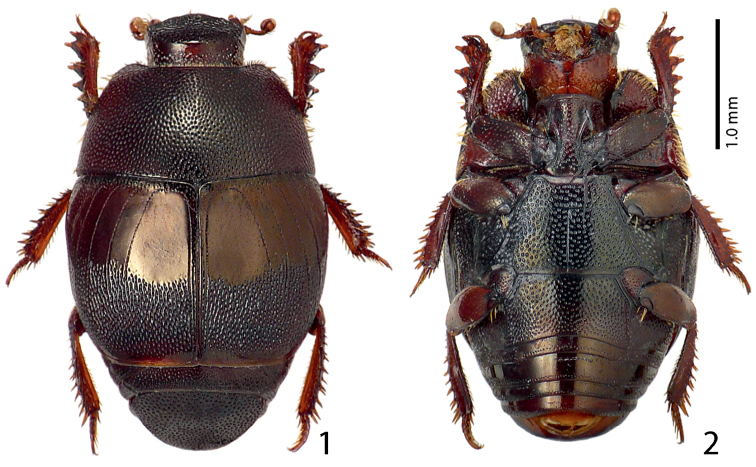
**1** *Sarandibrinus araceliae* gen. & sp. n., habitus, dorsal view **2** ditto, ventral view.

Figs 1 [Fig F2] [Fig F3] [Fig F4] [Fig F5] [Fig F6] –21

#### Type locality.

Madagascar, Toliara prov., Mahavelo Forest; Ifaty (Fig. 22).

#### Type material examined.

Holotype, ♂, side-mounted on a rectangular mounting card, male genitalia extracted and glued to the same card as specimen, with the following labels: “♂” (hand-written); followed by: “MADAGASCAR: Toliara / Prov., Forêt de Mahavelo / Isantoria River, elev 115 m / 5.5. km 37°NE Ifotaka / 31.i.2002” (printed); followed by: “25°45'13"S, 46°9'5"E / coll: Fisher, Griswold et al. / Calif. Acad. of Sciences / sifted litter - spiny forest / thicket, code: BLF5278” (printed); followed by: “CASENT / 8065358” (printed); followed by: “Sarandibrinus / araceliae sp. n. / Det. T. Lackner & Y. / Gomy 2014 HOLOTYPE” (red, hand-written label) (CAS). Paratypes: 1 ♂ & 4 ♀♀, same data, but: “CASENT / 8065359”; “CASENT / 8065357”; “CASENT / 8065361”; “CASENT / 8065360” (two female paratypes, “8065360” & “8065361” are sputter-coated with gold); 1 ♀, with the following labels: “MADAGASCAR: Prov. / Toliara; Ifaty / 23°09'S, 43°37'E / 17–22 Sept. 1993” (printed); followed by: “Flight intercept / yellow pan trap in / Malaise trap in / desert scrub forest” (printed); followed by: “W. E. Steiner & R. Andriamasimanana / collectors” (printed); followed by: “Sarandibrinus / araceliae sp. n. Det. / T. Lackner & Y. Gomy / 2014 PARATYPE” (red, hand-written label); 1 ♀, with the following labels: MADAGASCAR: "Toliara Prov. / Parc National d'Andohahela / Forêt de Manantalinjo 33.6 km / 63° ENE Amboasary, 7.6 km / 99° E Hazofotsy, 12-16 I 2002" (printed); followed by: "24°49'1"S, 46°36'36"E / coll: Fisher, Griswold et al. / California Acad. of Sciences / sifted litter - in spiny forest / thicket, elev. 150m BFL4810" (printed); followed by: "CASENT / 8065685" (printed); followed by: "Sarandibrinus / araceliae sp. nov. / Det. T. Lackner & / Y. Gomy 2014 PARATYPE" (red label, written); (1 ♂ in coll. CYG; 1 ♀ in coll. TLAN; rest of the paratypes in coll. CAS).

#### Description.

Body length: PEL: 2.20–2.50 mm; APW: 1.00–1.25 mm; PPW: 1.75–2.00 mm; EW: 1.75–2.20 mm; EL: 1.35–1.60 mm. Body (Figs 1–2) roundly oval, convex, elytra widest at humeri; cuticle of elytra castaneous, shining, without metallic luster; pronotum darker; body ventrally dark brown to almost black; abdominal ventrites (except for first visible) rufescent; legs, mouthparts and antennae light brown to castaneous.

**Figure 3. F2:**
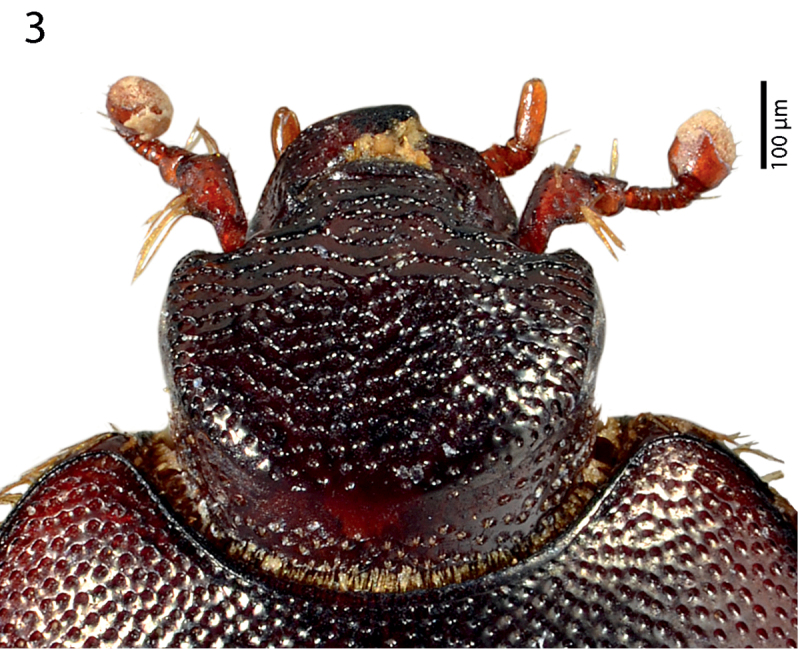
*Sarandibrinus araceliae* gen. & sp. n., head, dorsal view.

**Figures 4–11. F3:**
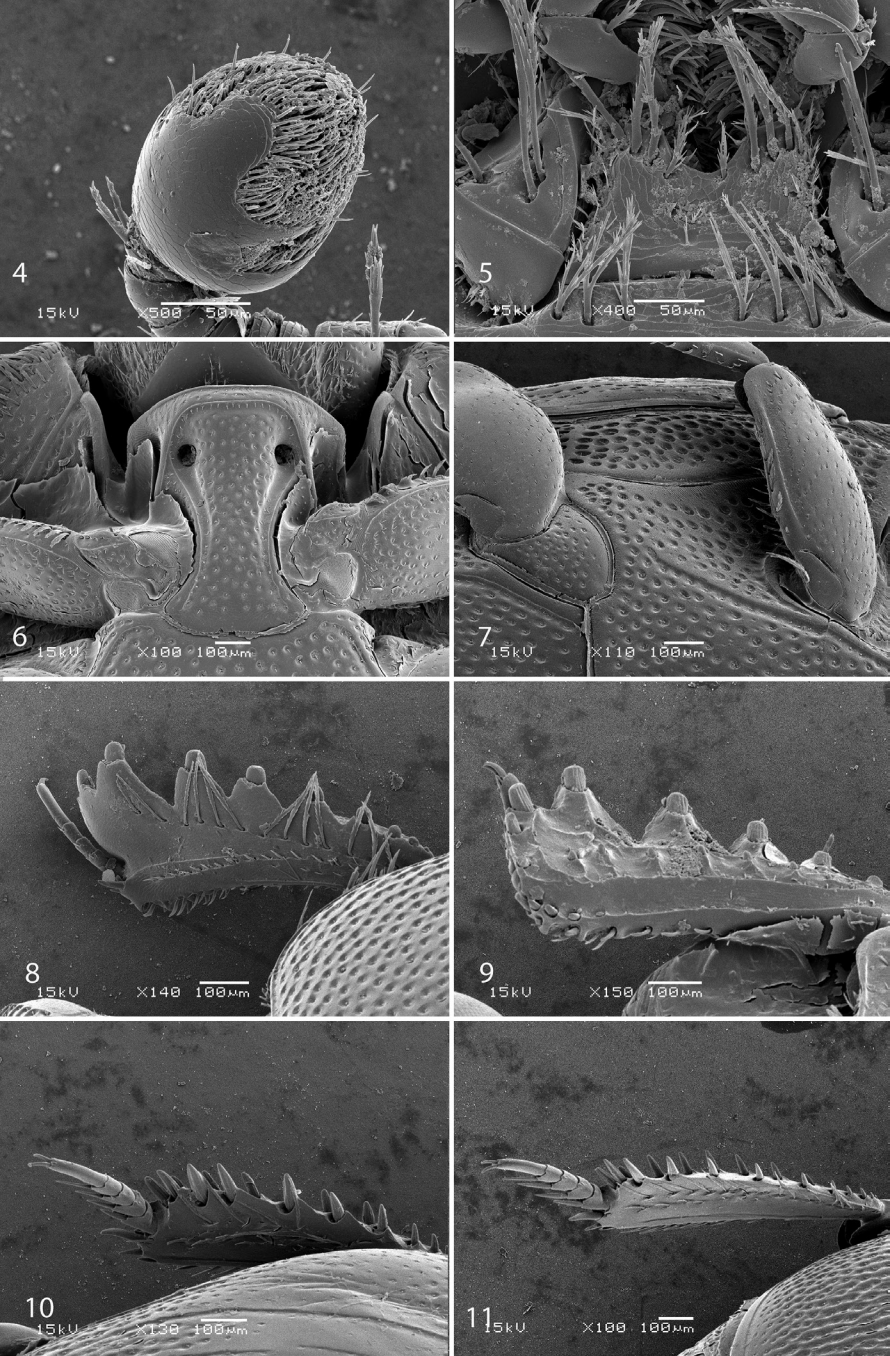
**4** *Sarandibrinus araceliae* gen. & sp. n., antennal club, ventro-lateral view **5** *Sarandibrinus araceliae* gen. & sp. n., mentum, ventral view **6** *Sarandibrinus araceliae* gen. & sp. n., prosternum **7** *Sarandibrinus araceliae* gen. & sp. n., lateral disk of metaventrite + metepisternum **8** *Sarandibrinus araceliae* gen. & sp. n., protibia, dorsal view **9** ditto, ventral view **10** *Sarandibrinus araceliae* gen. & sp. n., mesotibia, dorsal view **11** *Sarandibrinus araceliae* gen. & sp. n., metatibia, dorsal view.

Antennal scape (Fig. 3) slightly thickened, lower margin carinate, with several rather long ramose setae and several scattered punctures; club (Fig. 4) oval, without visible articulation, dorsal surface on basal 2/3 glabrous, apical 1/3 with dense short sensilla intermingled with sparser longer erect sensilla; ventrally (Fig. 4) setose patch covers larger part of antennal club as dorsally, on each (distal and proximal) side with a single slit-like orifice; sensory structures of antennal club in form a single ball-shaped vesicle situated on internal distal side of the antenna under the slit-like orifice (Fig. 13).

**Figure 12. F4:**
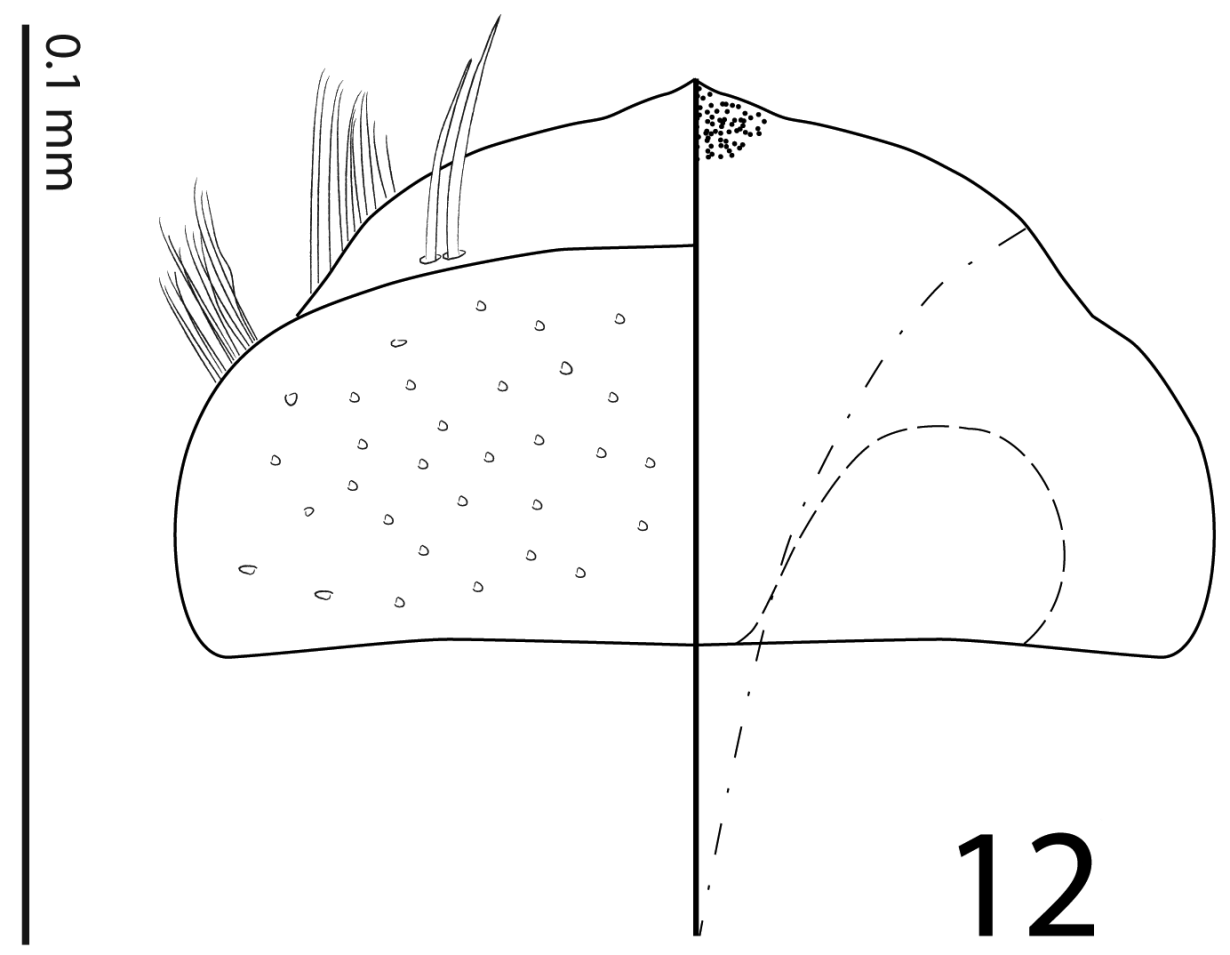
*Sarandibrinus araceliae* gen. & sp. n., labrum: left half depicting dorsal view and right half depicting epipharynx.

Mouthparts: mandibles obscurely variolate, punctate, mandibular apex pointed; sub-apical tooth of left mandible not examined; labrum (Fig. 12) convex, densely imbricate, its convexity interrupted by slight median concavity; labral pits situated near anterior margin, each with two well-sclerotized long setae; terminal labial palpomere thickened, about 1.5 times as long as penultimate, its width about half its length, truncate apically; mentum (Fig. 5) trapezoidal, anterior margin deeply inwardly arcuate, on each side with four long ramose setae, lateral margins with single row of much shorter ramose setae, disk of mentum imbricate; cardo of maxilla with few short setae; stipes triangular, with three long ramose setae; terminal maxillary palpomere thickened, truncate apically, about twice times as long as penultimate; its width about half its length.

**Figure 13. F5:**
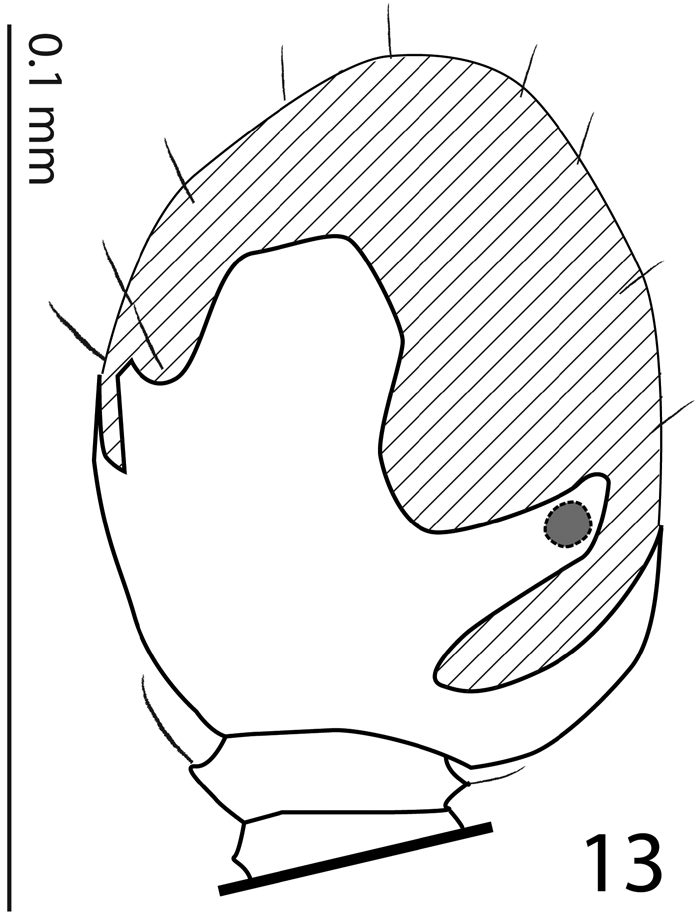
*Sarandibrinus araceliae* gen. & sp. n., antennal club, ventral view showing sensory structures of the antenna.

Clypeus (Fig. 3) rather short, flat, sub-quadrate, punctate, punctures linked with carinate strioles; frontal and supraorbital striae absent, in several specimens inter-linked carinate strioles appear as fragments of frontal stria; sculpture of frontal disc (Fig. 3) identical to that of clypeus; eyes flattened, almost invisible from above; occipital stria absent.

Pronotal sides (Fig. 1) moderately narrowing anteriorly; apical angles obtuse; anterior margin of pronotum broadly arcuate; pronotal depressions absent; marginal pronotal stria thin, slightly carinate and complete, somewhat weakened behind head; pronotal disc entirely covered by unusually deep and dense punctures, interspaces between them shorter than half their own diameter; pronotal hypomeron with fine dense yellow setae; scutellum well visible.

Elytral epipleura in several punctures; marginal epipleural stria fine, complete; marginal elytral stria straight, well impressed and slightly carinate, continued as weakened but complete apical elytral stria that is connected to complete sutural elytral stria. Humeral elytral stria short, deeply impressed on basal fourth, but difficult to discern due to very coarse and dense punctures and irregular strioles surrounding it; inner subhumeral stria present as a rather long medio-apical fragment; elytra with thin striae 1–4; striae in weak punctures, reaching approximately elytral half apically; fourth dorsal elytral stria basally connected with sutural elytral stria under broad arch; sutural elytral stria well-impressed and complete, in fine punctures, on apical half almost not discernible due to the extremely dense confluent punctation, apically connected with apical elytral stria, between it and elytral suture a row of fine punctures present; these fine punctures present also along entire elytral base and slightly entering elytral intervals basally; elytral humeri and flanks densely punctate, elytral disc on basal half (roughly) with large oval ‘mirror’ occupying approximately entire elytral intervals 1–4; apical half of elytra (roughly) covered with extremely coarse confluent punctures and longitudinal rugae, basally slightly entering also elytral intervals; extreme elytral apex impunctate.

Propygidium and pygidium densely and coarsely punctate, punctures separated by about their own diameter.

Anterior margin of median portion of prosternum (Fig. 6) almost straight; marginal prosternal stria present, almost complete; prosternal process between carinal prosternal striae slightly concave, with dense large setigerous punctation; carinal prosternal striae well-impressed, broadly divergent anteriorly, terminating in deep and large prosternal foveae; lateral prosternal striae carinate, sub-parallel, connected in front of apices of carinal prosternal striae, surface mesad of them with a regular row of fine microscopic setae.

Anterior margin of mesoventrite broadly inwardly arcuate; discal marginal mesoventral stria present only laterally, antero-medially obliterated; disc of mesoventrite with dense deep large punctures separated by less than their own diameter; meso-metaventral sutural stria absent; meso-metaventral suture well discernible; intercoxal disc of metaventrite in male with slight longitudinal median concavity almost indiscernible in female, completely covered by punctures identical to those of mesoventrite; lateral metaventral stria (Fig. 7) well impressed, carinate, almost straight, stopping short of metacoxa; lateral disc of metaventrite slightly concave, with punctation similar to that of metaventrite; metepisternum + fused metepimeron (Fig. 7) with even denser and deeper punctation; metepisternal stria absent.

Intercoxal disc of the first abdominal ventrite almost completely striate laterally; disc with punctation similar to that of lateral disc of metaventrite.

Protibia (Figs 8–9) slightly dilated, outer margin apically with single low tooth topped by denticle, followed by three large triangular teeth topped by denticle and another two low teeth topped by denticle; all five teeth diminishing in size gradually in proximal direction, followed by a three tiny denticles growing out directly from outer margin of protibia; setae of outer row regular, ramose, rather long; protarsal groove rather shallow; anterior protibial stria incomplete, bearing short and regular setae of intermedian row; another complimentary short stria originating approximately near tarsal insertion present; tarsal denticles absent; protibial spur (Fig. 8) short, straight, growing out from near the tarsal insertion; apical margin of protibia posteriorly with two widely separated tiny denticles; outer part of posterior surface with a sparse row of minute denticles situated on low protuberances, separated from imbricate median part of posterior surface by a definite ridge; posterior protibial stria complete, terminating in three tiny inner denticles; inner row of setae double, setae dense and short.

Mesotibia (Fig. 10) slender, outer margin with a dense row of approximately 7 prominent denticles situated inside low teeth; setae of outer row sparse, about as long as denticles themselves; setae of intermedian row shorter and finer than those of outer row, regular; posterior mesotibial stria almost complete; anterior surface of mesotibia variolate-punctate, with another row of approximately 7 denticles shorter than those of outer row; anterior mesotibial stria complete, terminating in single tiny inner anterior denticle; mesotibial spur rather long; apical margin of mesotibia with three short denticles; mesotarsomeres telescopically becoming narrower apically, each bearing two thick setae ventrally; claws of apical tarsomere slightly bent, approximately one-third its length; metatibia (Fig. 11) slenderer and longer than mesotibia, in most respects similar to it, but denticles of outer margin not growing out from inside low teeth and setae of outer row shorter than those of mesotibia.

**Figures 14–20. F6:**
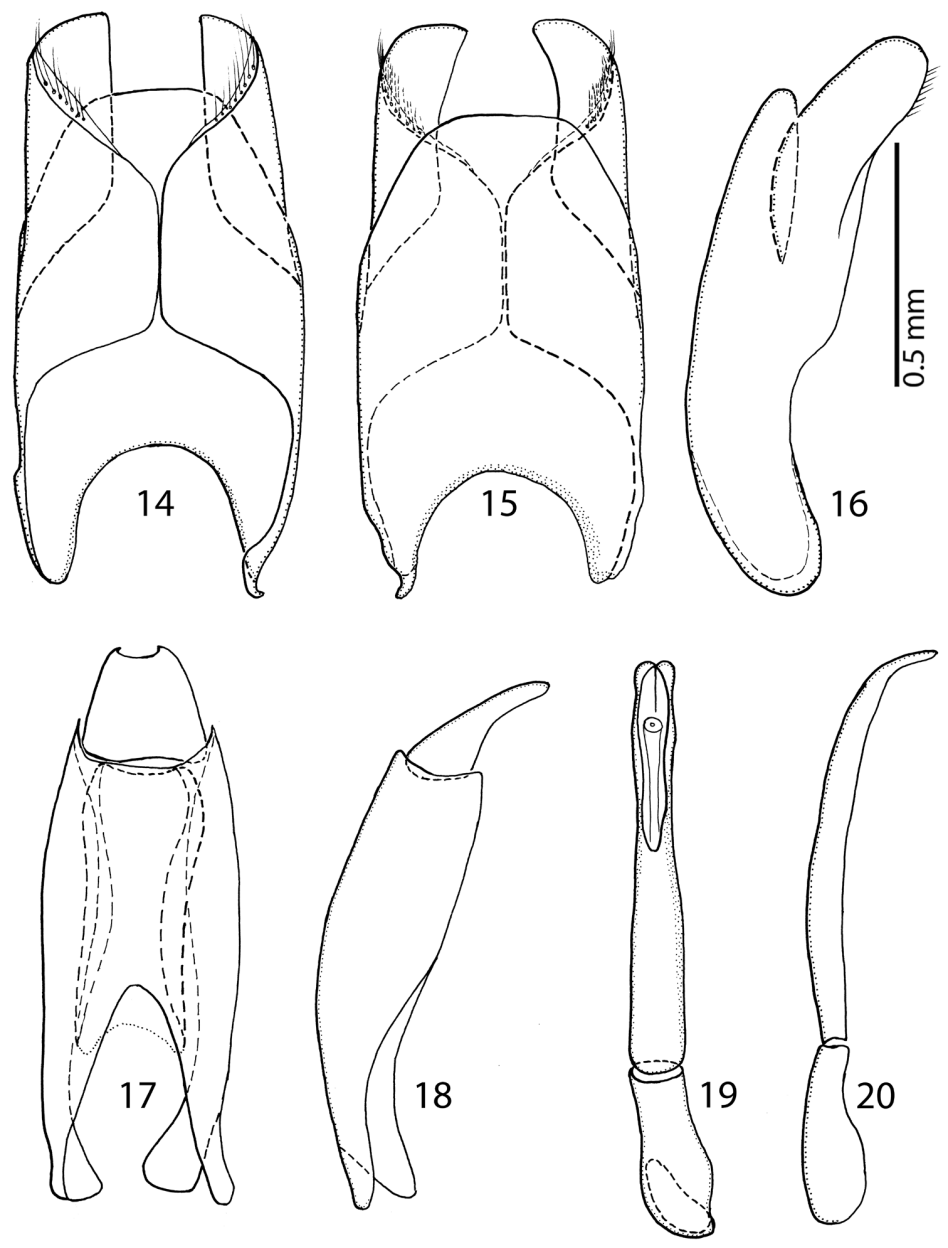
**14** *Sarandibrinus araceliae* gen. & sp. n., male terminalia: 8^th^ sternite and tergite, ventral view **15** ditto, dorsal view **16** ditto, lateral view **17** *Sarandibrinus araceliae* gen. & sp. n., male terminalia: 9^th^ + 10^th^ tergites, dorsal view; spiculum gastrale, ventral view **18** *Sarandibrinus araceliae* gen. & sp. n., male terminalia: 9^th^ + 10^th^ tergites, lateral view **19** *Sarandibrinus araceliae* gen. & sp. n., male terminalia: aedeagus, dorsal view **20** ditto, lateral view.

**Figure 21.**
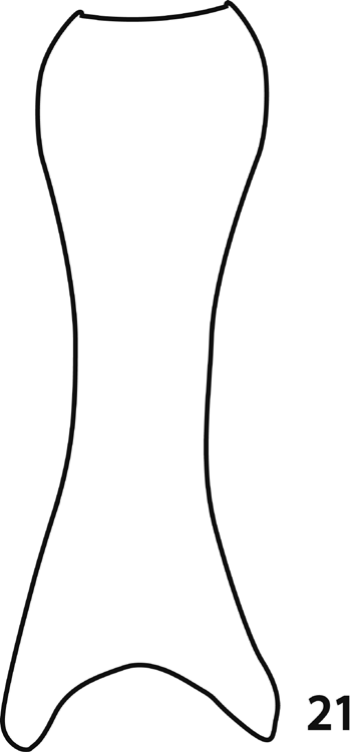
*Sarandibrinus araceliae* gen. & sp. n., male terminalia: reconstruction of the spiculum gastrale, ventral view.

Male genitalia. Eighth sternite (Figs 14–15) apically with tiny vela adorned with a row of long, sparse setae; halves separated medially. Eighth tergite (Fig. 15) apically outwardly arcuate; eighth sternite and tergite fused laterally (Fig. 16). Ninth tergite (Figs 17–18) when compared to tenth tergite conspicuously long, acutely inwardly arcuate basally; tenth tergite (Fig. 17) apically slightly inwardly arcuate. Spiculum gastrale of both known male specimens (on basal part) damaged during the manipulation with genitalia, so we are only able to depict its ‘reconstruction’ here (Fig. 21): median part only slightly constricted, typical ‘head’ and ‘stem’ (sensu Caterino and Tishechkin 2013) absent. Aedeagus (Figs 19–20) with rather large basal piece, its length ratio to the length of the parameres approximately 1:3. Aedeagus on the whole slender; parameres of the aedeagus fused on the basal half (approximately), aedeagus constricted medially and laterally curved.

#### Etymology.

This species is dedicated to Mrs. Araceli Cancino, wife of the famous Belgian comics illustrator Midam, in appreciation of the publication of the three volumes of “Carnets de Grrreeny”.

### New records on the distribution of the Saprininae from Madagascar and Mauritius

*Hypocaccus (Baeckmanniolus) rubiciliae* (Lewis, 1899)

**Figure 22.**
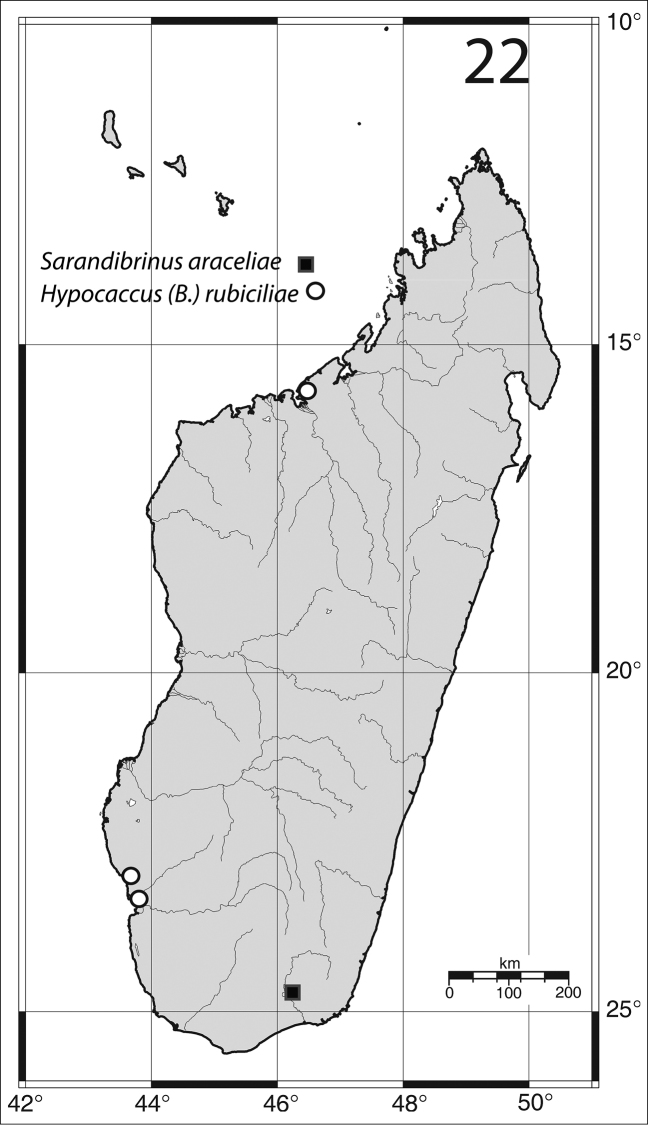
Distribution of *Sarandibrinus araceliae* gen. & sp. n. and *Hypocaccus (Baeckmanniolus) rubiciliae* Lewis, (1899) on the Island of Madagascar.

Described from Tanzania, reported also from the Republic of South Africa (Natal) (Mazur 2011). New to Madagascar (Fig. 22).

Madagascar south-west: 21 exs., Toliara, “Plage de La Batterie”, 19.vii.1969, under algae (Y. Gomy leg.). 5 exs., Toliara env., “Fitsitika”, 20.vii.1969, beach, under algae (Y. Gomy leg.). Madagascar North: 22 exs., Majunga, “Village touristique”, 29.VII.1968, beach, under coastal wrack (Y. Gomy leg.).

*Hypocaccus (Nessus) perparvulus* (Desbordes, 1916)

Described from Madagascar, new to Mauritius.

Mauritius, 1 ex., Pointe aux Sables, 15.i.1971, in a coop (Y. Gomy leg.). Gomy (1983) erroneously reported this specimen under the name *Hypocaccus (Nessus) grandini* (Marseul, 1870).

### Key to the genera of the Saprininae of Madagascar

(For the sake of wider usage this key contains also taxa known to occur on the nearby archipelagos of Mascarene Islands, Comoros and Seychelles.)

**Table:** 

1 (6)	Head without any trace of frontal or supra-orbital striae (Fig. 3)
2 (3)	Frons very densely and coarsely punctate (Fig. 3); punctures forming elongate rugae	*Sarandibrinus* gen. n.
3 (2)	Frons with fine, scattered punctation; punctures never forming elongate rugae (Fig. 23)
4 (5)	Carinal prosternal striae joined anteriorly (Fig. 24); elytra with characteristic short hooked appendix between fourth dorsal and sutural elytral striae, marginal elytral stria double	*Gnathoncus* Jacquelin Du Val, 1858
5 (4)	Carinal prosternal striae divergent anteriorly (Fig. 25), in some cases joined by a deep sulcus; elytral disk between fourth dorsal elytral and sutural elytral striae without a hooked appendix; marginal elytral stria single	*Euspilotus* Lewis, 1907
6 (1)	Frontal and/or supra-orbital striae present (Fig. 26)
7 (8)	Prosternal foveae absent (Fig. 27)	*Saprinus* Erichson, 1834
8 (7)	Prosternal foveae present (Fig. 28)
9 (10)	Frontal stria widely interrupted medially, prolonged onto clypeus (for fig. see Lackner and Gomy 2013, fig. 6). Prosternal foveae oblique, large and deep (for fig. see Lackner and Gomy 2013, fig. 4). Punctation of pronotum and elytra very coarse and dense, forming elongate rugae overall	*Malagasyprinus* Lackner & Gomy, 2013
10 (9)	Frontal stria complete (Fig. 29), prosternal foveae small to moderately large (Fig. 28), never deep; punctation of dorsum never forming elongate rugae
11 (12)	Frons (Fig. 29) with distinct punctation, without obvious chevrons or transverse rugae. Body size from 1.50–2.00 mm	*Hypocacculus* Bickhardt, 1914
12 (11)	Frons (Fig. 30) smooth with chevrons or with more or less regular transverse rugae. Body size usually larger than 2.00 mm	*Hypocaccus* Thomson, 1867

**Figures 23–30.**
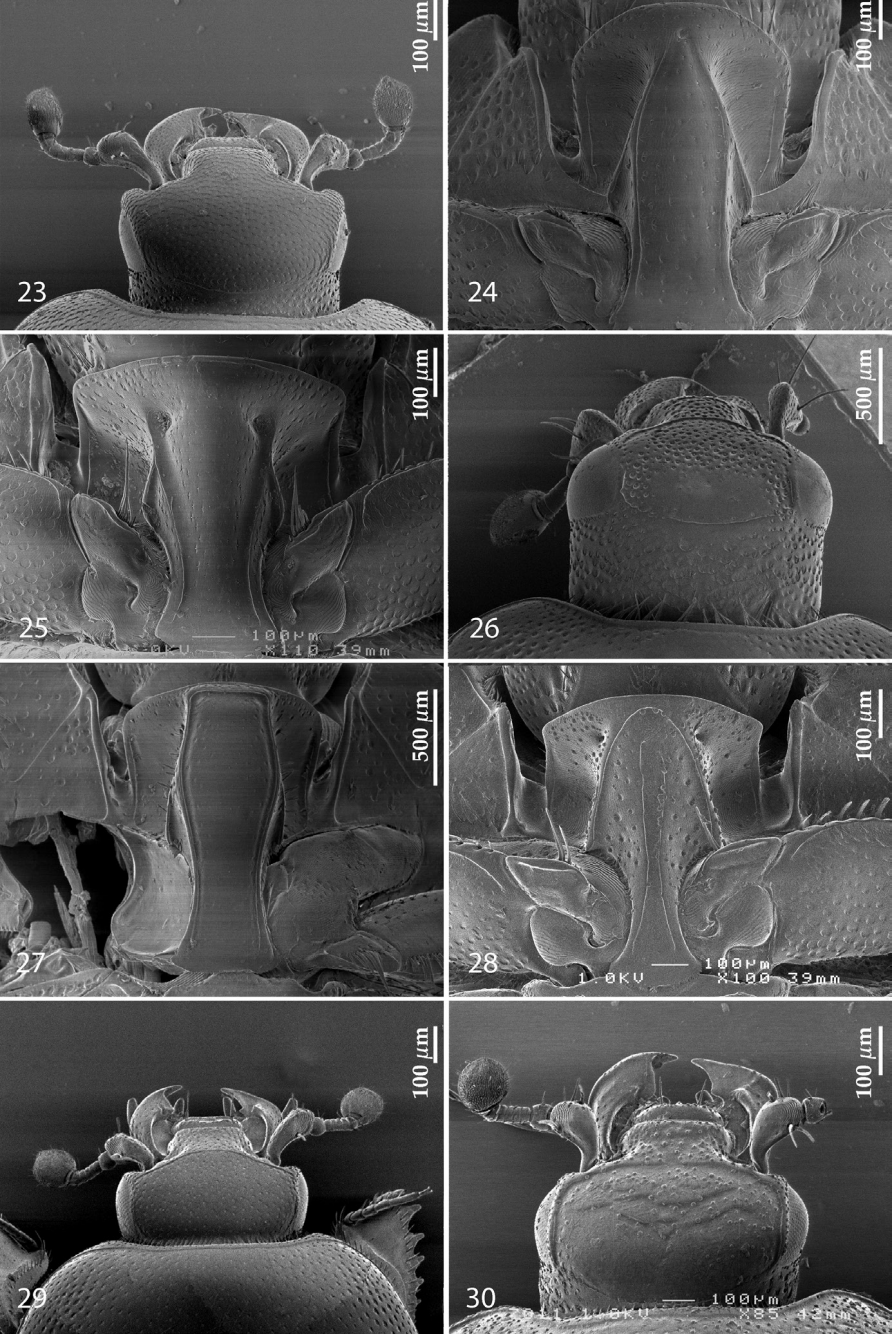
**23** *Euspilotus (Neosaprinus) rubriculus* (Marseul, 1855), head, dorsal view **24** *Gnathoncus rotundatus* (Kugelann, 1792), prosternum **25** *Euspilotus (Hesperosaprinus) modestus* (Erichson, 1834), prosternum **26** *Saprinus (Saprinus) fulgidicollis* Marseul, 1855, head, dorsal view **27** ditto, prosternum **28** *Hypocaccus (Hypocaccus) brasiliensis* (Paykull, 1811), prosternum **29** *Hypocacculus (Hypocacculus) metallescens* (Erichson, 1834), head, dorsal view **30** *Hypocaccus (Hypocaccus) brasiliensis* (Paykull, 1811), head, dorsal view.

## Discussion

The newly erected genus exhibits a mixture of plesiomorphic and apomorphic characters. Among the plesiomorphic ones (sensu Lackner, unpublished) is undoubtedly the absence of both supra-orbital and frontal striae; these striae are without exception absent with the taxa near the root of the recently performed phylogenetic analysis aimed at resolving the relationships of the higher taxa of the Saprininae subfamily (Lackner, unpublished).

The presence of large, deep oblique prosternal foveae is undoubtedly an apomorphic character (as it is present mainly among more ‘derived’ Saprininae; see also Discussion in Lackner and Gomy 2013 for details), as is the single ball-shaped vesicle present inside the antennal club. A prosternum with such large and deep oblique prosternal foveae is found only in another Malagasy endemic, genus *Malagasyprinus* Lackner & Gomy, 2013, which makes their inter-relationships intriguing. We have included both taxa in the matrix of morphological characters of the higher taxa of the Saprininae subfamily developed recently by the senior author and our analyses placed them distant from each other, which suggests that they most likely do not share a common ancestor. Both taxa were, however, members of a large clade consisting of taxa that share a single, pear-shaped vesicle inside their antennal club. Relationships among members of this clade were mostly unresolved and they represent taxa mostly spread in the Old World, with several psammophilous members described from North America (Lackner, unpublished). The number of vesicles inside the antennal club among the Saprininae tends to decrease with the more ‘derived’ taxa, while the taxa placed near the root of the tree (Lackner, unpublished) usually possess more than two vesicles inside their antennal clubs. However, the presence of a single vesicle, which is a synapomorphy of the true ‘derived’ Saprininae is always pear-shaped regarding its shape, whereas the taxa placed near the root of the cladogram have their vesicles always ball-shaped (although usually numerous; Lackner, unpublished). From this respect the vesicle character in *Sarandibrinus* is ambiguous, as it is single in number, but ball-shaped instead of pear-shaped.

The curiously shaped spiculum gastrale (Fig. 21), resembling that of a monotypic Namibian endemic *Paraphilothis mirabilis* Vienna, 1994 (Lackner, unpublished) presents an interesting question here, seen from the phylogenetic standpoint. It is likely that such a structure evolved twice independently within the Saprininae subfamily, as the two taxa *Sarandibrinus* and *Paraphilothis* are not related according to our analyses. *Paraphilothis mirabilis* was recovered closer to the root of the cladogram, distant from the above-mentioned clade of taxa possessing a single pear-shaped vesicle in their antennal club. *Paraphilothis* has been observed to possess four ball-shaped vesicles inside its antennal club (Lackner, unpublished). Emerging ideas of the higher phylogeny of the Saprininae subfamily suggest that there remains still a significant work to be performed.

Last, but not least, we should probably mention the unusual collecting conditions of this new taxon. Although Saprininae are very plastic in their ecologies (Lackner, forthcoming), there has not been so far any record of collecting a member of the Saprininae by the method of litter sifting or yellow pan trapping. It is obvious that the island of Madagascar, known for its high endemism among the living organisms has still a lot to offer from its store.

## Supplementary Material

XML Treatment for Sarandibrinus

XML Treatment for Sarandibrinus araceliae
